# Supplementary material for: Protracted Functional and Structural Reorganization of Human Prefrontal Cortex Supports Lateralized Category Geometries
Source: bioRxiv. 2026 May 6:2026.05.04.721346. Preprint. [Version 1] doi: 10.64898/2026.05.04.721346 (PMC13174603; doi:10.64898/2026.05.04.721346)
Supplement: 1 [file NIHPP2026.05.04.721346V1-supplement-1.pdf]

bioRxiv preprint doi: https://doi.org/10.64898/2026.05.04.721346; this version posted May 6, 2026. The copyright holder for this preprint (which was not certified by peer review) is the author/funder, who has granted bioRxiv a license to display the preprint in perpetuity. It is made available under aCC-BY 4.0 International license.

Supplemental Information

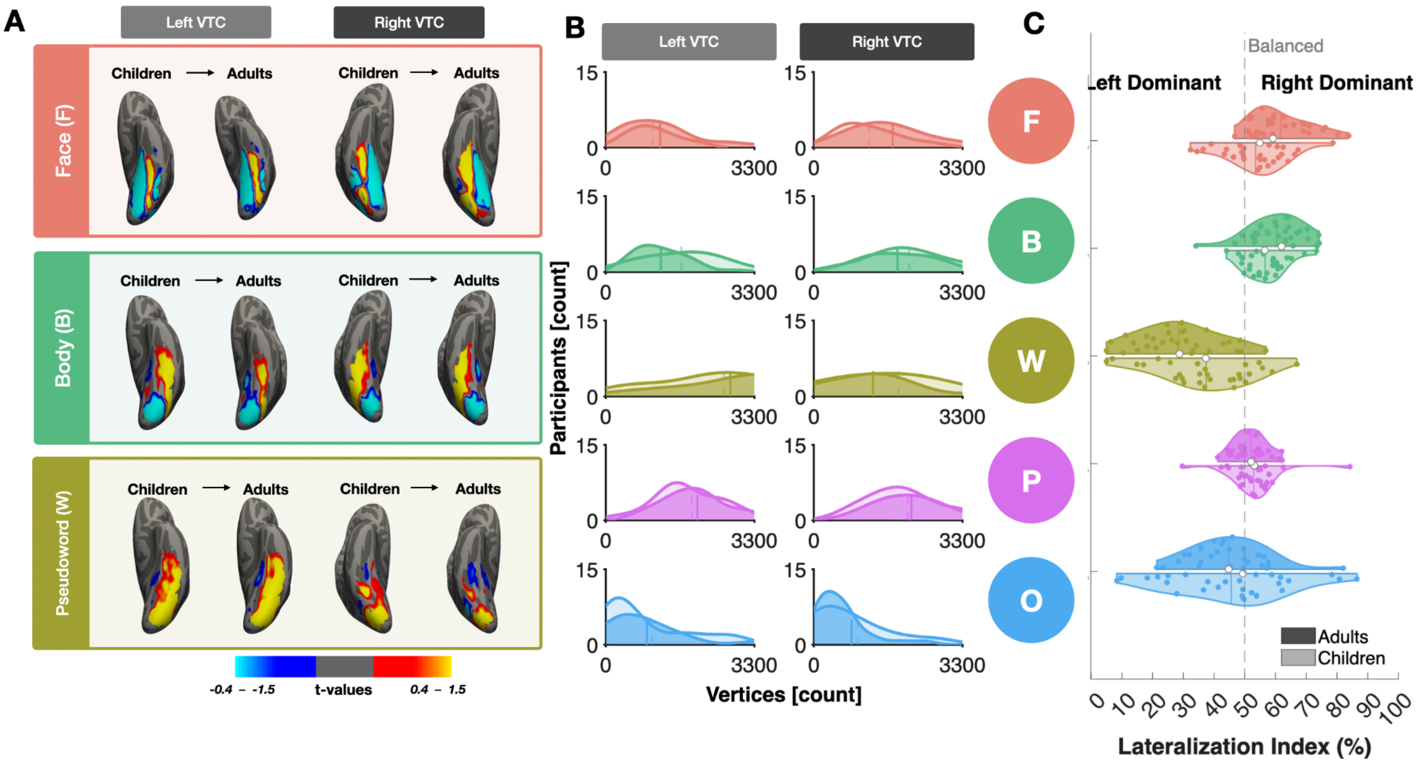

***Supplementary Figure 1. VTC shows robust, canonical category-selective organization. (A)** Average cortical surface maps of category selectivity (preferred > all others) for faces, bodies, and words on the fsaverage template. Columns show a sample stimulus, left hemisphere maps, and right hemisphere maps; each hemisphere panel shows children (left) and adults (right). Diverging colors indicate positive (red–yellow) and negative (blue) selectivity. **(B)** Density plots of suprathreshold vertex counts (t-values > 2.5) for five visual categories in each hemisphere; translucent and solid curves indicate children and adults, respectively; vertical lines show group means. **(C)** Split-violin plots of hemispheric dominance (% RH activation relative to LH + RH) per category for children (lighter) and adults (darker); values above and below 50% indicate RH and LH dominance, respectively.*

bioRxiv preprint doi: https://doi.org/10.64898/2026.05.04.721346; this version posted May 6, 2026. The copyright holder for this preprint (which was not certified by peer review) is the author/funder, who has granted bioRxiv a license to display the preprint in perpetuity. It is made available under aCC-BY 4.0 International license.

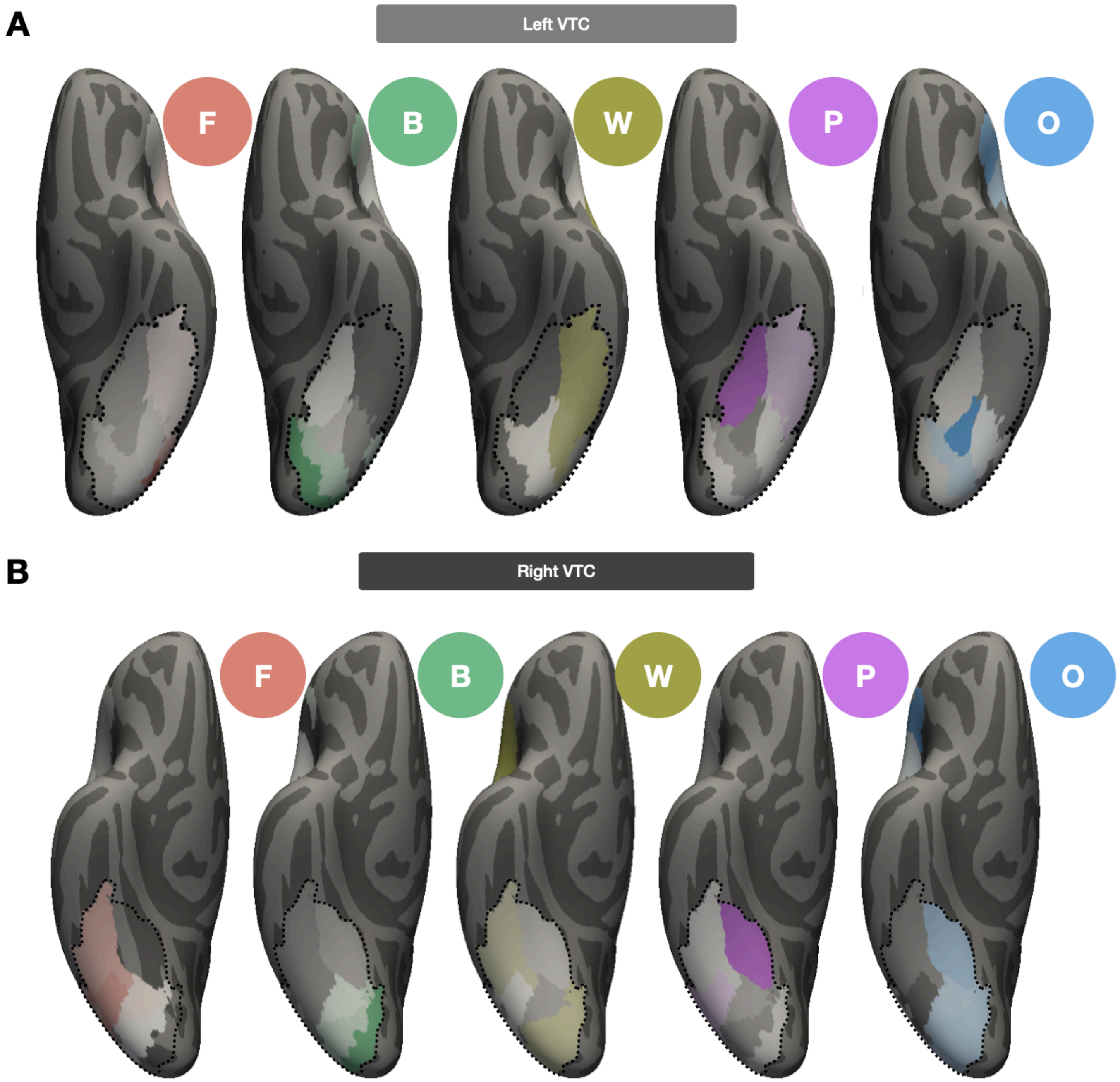

Supplementary Figure 2. VTC category response patterns show modest developmental differences relative to VLPFC. (A) *Z-scored developmental difference maps (adults minus children, normalized within each system) projected onto LH VTC surface. Positive/negative values indicate stronger adult/child responses.* ***(B)*** *Same as A for RH VTC.*

bioRxiv preprint doi: https://doi.org/10.64898/2026.05.04.721346; this version posted May 6, 2026. The copyright holder for this preprint (which was not certified by peer review) is the author/funder, who has granted bioRxiv a license to display the preprint in perpetuity. It is made available under aCC-BY 4.0 International license.

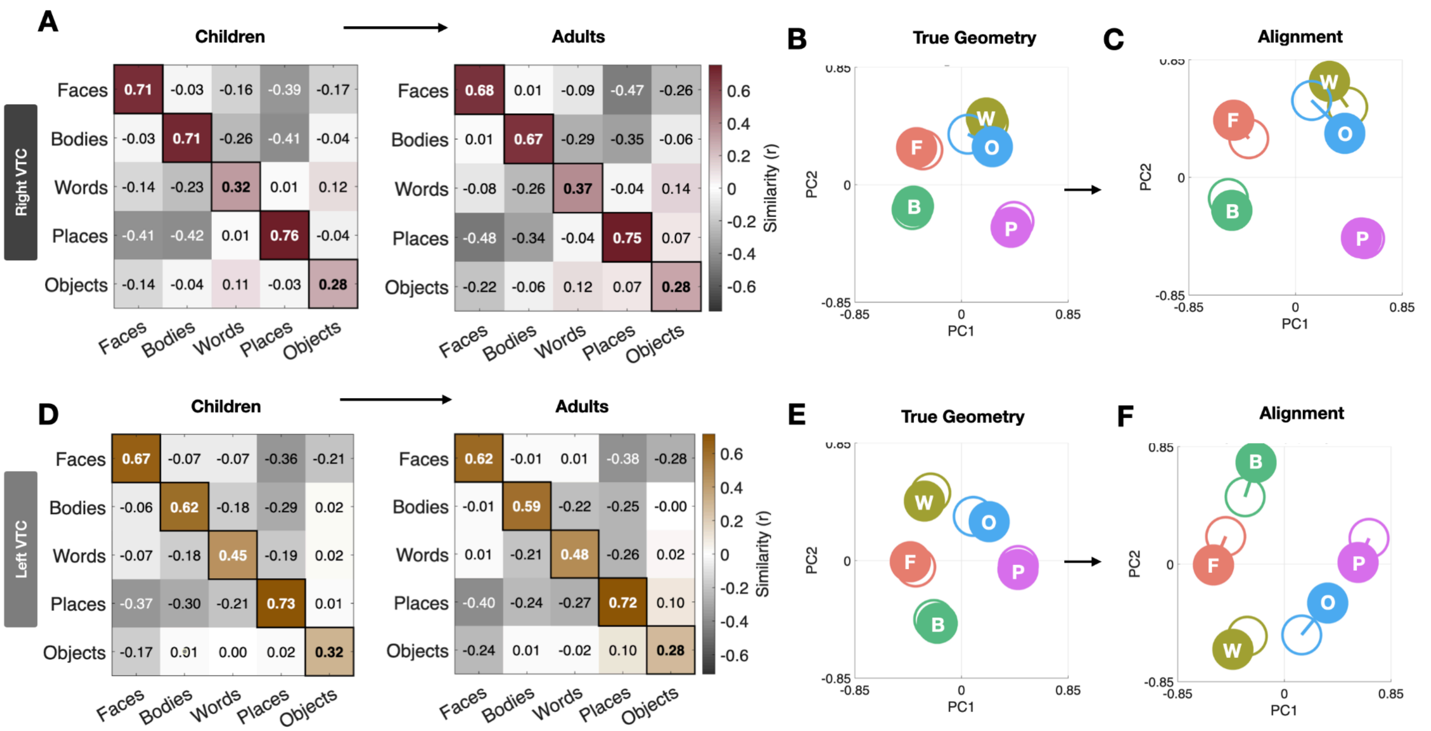

***Supplementary Figure 3. VTC representational geometry is stable across development. (A)** LH VTC representational similarity matrices (mean pairwise Pearson r between voxelwise t-value maps for five categories) computed using LORO cross-validation, shown separately for children and adults. Diagonal = within-category reliability; off-diagonal = cross-category similarity. **(B)** PCA of voxelwise category-selective response patterns in LH VTC, plotted in PC1–PC2 space (z-scored across vertices). Filled/open circles = adults/children; lines connect matched categories across groups. **(C)** Same as B, following Procrustes alignment of children to the adult configuration. **(D–F)** Same as A–C for RH VTC.*
